# Comparing Implicit and Explicit Attitudes Toward Intimate Partner Violence Against Women

**DOI:** 10.3389/fpsyg.2020.02147

**Published:** 2020-09-08

**Authors:** Victoria A. Ferrer-Perez, Esperanza Bosch-Fiol, Virginia Ferreiro-Basurto, Carmen Delgado-Alvarez, Andrés Sánchez-Prada

**Affiliations:** ^1^Faculty of Psychology, University of Balearic Islands, Palma, Spain; ^2^Faculty of Psychology, Pontifical University of Salamanca, Salamanca, Spain

**Keywords:** intimate partner violence against women, attitudes, implicit assessment, explicit assessment, implicit association test

## Abstract

Intimate partner violence against women (IPVAW) is an epidemic social and public health problem. Research has consistently found evidence for a complex etiology of IPVAW resulting from the interaction of many factors, among which gender-related norms and attitudes are among the main drivers of this violence. Public attitudes toward IPVAW are especially important because attitudes rejecting, condoning, or fostering such behavior are social factors that contribute to a climate of tolerance or refusal that can shape the social environment in which such violence takes place. Given the importance of these attitudes, the availability of reliable, valid, and concise measures is critical for both research and intervention purposes. The evidence shows a probable bias of direct or explicit assessment measures of IPVAW attitudes, and it has been suggested that they should be complemented by indirect or implicit measures. In this context, the main aim of this paper was to examine how implicit and explicit attitudes toward IPVAW differ among a Spanish population. An opportunity sample of 693 students took part in this study. Two direct or explicit measures (the Inventory of Distorted Thoughts about Women and Violence, IPDMV, and the Inventory of Beliefs about Intimate Partner Violence, IBIPV) and one indirect or implicit measure [the Gender Violence Implicit Association Test (GV-IAT), a personalized form of the Implicit Association Test (IAT)] were applied. The results obtained show that the psychometric characteristics of the implicit measure used (GV-IAT) are acceptable. Additionally, we obtained significant differences by gender, IPVAW knowledge, IPVAW involvement, and political opinion when we measured IPVAW attitudes by implicit measures. However, when we measured these attitudes by explicit measures, we only obtained significant differences by gender and political opinion. Finally, the results highlight the important differences between the levels of strong IPVAW rejection measured with explicit and implicit measures, confirming the traditional discrepancy between explicit and implicit measures of attitudes. In summary, the results obtained provide additional support for the idea that GV-IAT constitutes a promising assessment tool to complement explicit measures for attitudes toward IPVAW.

## Introduction

Intimate partner violence against women (IPVAW) is a violation of women’s human rights and an epidemic social and public health problem (World Health Organization [WHO], 2013), which can be said to “interfere with their full participation in society and in their countries’ social and economic development” (Heise and Kotsadam, 2015, p. e332).

In relation to its prevalence, IPVAW is “one of the most common forms of violence experienced by women” (Devries et al., 2013, p. 1527), although research shows that its prevalence differs across countries and regions (Heise and Kotsadam, 2015). For instance, in Europe, a survey conducted in the 28 states of the European Union, with similar questions and methods among the countries, estimated that an average of 22% of European women have been victims of IPVAW since the age of 15, with rates across countries ranging from 13% (Spain) to 32% (Denmark). The 12-month prevalence varies from 2% in countries such as Spain, Estonia, or Slovenia to more than 6% of women in countries such as Italy, Greece, or Bulgaria (European Union Agency for Fundamental Rights [EUAFR], 2014). In the same vein, the Global Health Observatory data repository (2019) estimated an IPVAW prevalence of 19.3% in Western Europe.

With regard to its etiology, research has consistently found evidence for a complex etiology of IPVAW resulting from the interaction of many factors, and indeed, different theories exist about their causes (Dixon and Graham-Kevan, 2011). Feminists have long contended that gender-related norms and attitudes are among the main drivers of IPVAW, including those accepting and justifying wife beating, the hierarchical relationships between men and women, male superiority and authority over females, or practices and laws that disadvantage women in access to productive resources (Heise and Kotsadam, 2015). Some research confirms that these factors are “highly predictive of rates of perpetration and victimization” (Sardinha and Najera, 2018, p. 2) and, consequently, of the geographical distribution of IPVAW (Archer, 2006; Heise and Kotsadam, 2015; Puente et al., 2016; Sugarman and Frankel, 1996). In this theoretical context, it may be pointed out that public attitudes toward IPVAW are especially important because “attitudes condoning or fostering partner violence are social factors that contribute to a climate of tolerance that may influence incidence rates” (Gracia and Tomás, 2014, p. 26) or that can shape the social environment in which such violence takes place. In other words, these factors either foster or discourage IPVAW in societies and, consequently, directly impact the extent of perpetration, incidence rates, and reporting rates of this type of violence, along with public and professional perceptions and responses to it, and the victims’ own responses and help-seeking behaviors (Flood and Pease, 2009; Gracia, 2014; Gracia and Tomás, 2014; Gracia et al., 2014, 2015; Rizo and Macy, 2011; Wang, 2016).

To begin, supportive attitudes of IPVAW, such as acceptability or tolerance towards IPVAW, are still widespread and have usually been linked to the perpetration of this type of violence, given that high levels of IPVAW acceptability can conduct to perceive this violence as normative, increasing the risk perpetration by men, and the risk of justification by victims and social context (Martín-Fernández et al., 2018), which in turn increase the risk of this occurrence (Sugarman and Frankel, 1996; Fincham et al., 2008; Flood and Pease, 2009; Capaldi et al., 2012; Gracia et al., 2015; Wang, 2016; Herrero et al., 2017; Copp et al., 2019; Romero et al., 2019).

Secondly, “acceptability of IPVAW is also related to the kind of behavior that is considered violent in intimate relationships” (Gracia et al., 2015, p. 2). If some IPVAW episodes are perceived as less serious, more acceptable, or even deserved by the victims who are blamed or considered responsible for provoking violence, this may increase not only the perpetration of violence by men with a history of IPVAW but also its legitimization by the victims themselves and their social environment (Waltermaurer, 2012; Gracia, 2014; Gracia et al., 2015).

Thirdly, having a positive attitude toward IPVAW can put women at risk of suffering this violence (Wang, 2016). Thus, the extent to which a female victim of IPVAW agrees with violence-supportive beliefs is directly related to the likelihood of her blaming herself for the violence suffered. She may also be reluctant to report this violence, to seek support, or to leave the relationship, as she may think that “their social circle accepts this violence or considers it justified, therefore leading victims (her) to consider help as less likely or deserved” (Gracia et al., 2015, p. 2) and to be more likely to experience negative and serious psychosocial and emotional consequences (Flood and Pease, 2009).

Finally, attitudes toward IPVAW determine also the responses to this violence shown by other people such as family members, friends, professionals, or bystanders (Flood and Pease, 2009). Thus, individuals with more violence-supportive attitudes respond by showing less empathy and support to victims, blaming them, avoiding reporting the incidents, and denying the penalties for men with a history of IPVAW (Valor-Segura et al., 2011; Gracia and Tomás, 2014; Eigenberg and Policastro, 2016; Meyer, 2016).

In summary, “the evidence available shows that a climate of acceptance and tolerant beliefs, and attitudes toward IPVAW, constitutes some of the most important socio-cultural risk factors for its occurrence (and) these concepts are present in many of the multi-causal models used to explain it nowadays” (Ferrer and Bosch, 2014, 508).

Related to the prevalence of supportive attitudes toward IPVAW, several demographic surveys have included brief scales or individual items measuring IPVAW acceptability (European Commission, 2010; Yount et al., 2011; World Health Organization [WHO], 2013; European Union Agency for Fundamental Rights [EUAFR], 2014; Gracia and Lila, 2015; Herrero et al., 2017; Schwab-Reese and Renner, 2017; Sardinha and Najera, 2018; Tausch, 2019; Vázquez et al., 2019). For instance, the 2010 Eurobarometer (European Commission, 2010) showed that in the European Union, an average of 84% of those surveyed considered that IPVAW was unacceptable and should always be punishable by law (with variations across countries from 93% to 66%) (Gracia and Lila, 2015); 12% of respondents believed that IPVAW was unacceptable but not always punishable by law, also with substantial variation across countries (i.e., from a low of 5% in Greece to a high of 32% in Finland); and only a very small fringe of those interviewed thought that IPVAW was acceptable in certain circumstances (2%) or in all circumstances (1%). This survey also included a question tapping into victim-blaming attitudes, asking whether the provocative behavior of women was a cause of domestic violence, with the percentage of people agreeing with this statement ranging from 33% (in Spain) to 86% (in Lithuania) and an EU average of 52%. In summary, these results show that the acceptability of IPVAW, and a victim-blaming attitude in particular, is still widespread, creating a social climate that, to some extent, condones IPVAW.

Herrero et al. (2017) analyzed a large dataset of respondents from the World Values Survey (WVS) in 51 countries around the world providing information about sexism and the acceptability of IPVAW and general violence in social relationships. Their results showed that both were positively related to acceptability, but the “highest levels of acceptability of IPV were found among sexist individuals who also present positive attitudes toward the use of violence in social relationships” (p. 351).

Additionally, Tausch (2019) analyzed data from the WVS, specifically data from 72 countries around the world, and observed that some Western European countries (such as Italy or Norway) are among those with the lowest rates of acceptance of IPVAW.

Other studies have developed and applied attitudinal scales to measure the acceptability of IPVAW among different European specific populations, such as adolescents (Smith et al., 2005; Fincham et al., 2008; Copp et al., 2019; Fernández et al., 2017), professionals (Djikanovic et al., 2010) or men with a history of IPVAW (Echeburúa and Fernández-Montalvo, 1998), and also among the general population (Saunders et al., 1987; Martín-Fernández et al., 2018).

Despite these examples, the review administered by Gracia and Lila (2015) of the research on attitudes toward IPVAW in the European Union found that (i) only a relatively limited number of surveys conducted among the EU Member States between 2010 and 2014 (40 surveys in 19 countries) included questions addressing attitudes toward violence against women (mostly related to IPVAW) and (ii) in general, available information from these surveys was scarce, mostly based on single items, and not supported by instruments with enough evidence of validity and reliability. Additionally, it is worth pointing out that almost all of this research was carried out with self-reports or direct or explicit assessment measures of attitudes, which “often provide distorted or socially desirable accounts that may misrepresent respondents’ actual attitudes” (Gracia et al., 2015, p.1).

Related to their predictors, attitudes toward IPVAW are influenced by different types of factors (Flood and Pease, 2009). In this regard, studies have frequently indicated several sociodemographic factors influencing attitudes concerning the acceptability of IPVAW, with variations in these attitudes accounted for by gender, age, education, residency, income or economic status, marital status, or residence (Waltermaurer, 2012; Gracia and Tomás, 2014; Wang, 2016; Herrero et al., 2017).

Gender is one of the most consistent predictors of supportive attitudes toward IPVAW (Flood and Pease, 2009), and in general, research confirms a gender gap in these attitudes (Gracia et al., 2015; Martín-Fernández et al., 2018; Sardinha and Najera, 2018) from an early age (Ferragut et al., 2017): women and girls show lower levels of IPVAW acceptance than men, and men and boys have a greater tendency to agree with myths and supportive attitudes toward IPVAW, blame women victims for the violence suffered and show less empathy for them, and consider violent incidents as less serious or damaging and minimize them (Flood and Pease, 2009). It is also important to point out, as do Flood and Pease (2009), that it is gender orientations (not sex) that explain men’s and women’s different understandings of IPVAW.

Age and age-developmental processes are another factor shaping attitudes toward IPVAW (Flood and Pease, 2009). In this regard, “younger individuals might be expected to have more informed attitudes toward IPVAW, reflecting improvements over time in attitudes as well as the influence of their generation’s greater exposure to university and other positive influences” (Flood and Pease, 2009, p. 132), while “older generations tend to hold more traditional views regarding the acceptance of violence as a normal part of relationships, as IPVAW was not a social issue when they reached their adulthood” (Gracia and Tomás, 2014, p. 35). Indeed, some studies show that attitudes supporting IPVAW tend to be more prevalent among older people (Gracia and Tomás, 2014; Gracia et al., 2015). On the other hand, others show that younger participants justified wife beating more often (Rani and Bonu, 2009), a pattern that might have at least three explanations (Flood and Pease, 2009, p. 132): a greater endorsement among younger boys of this violence may reflect their lack of exposure to the liberalizing influence of education; it may reflect developmental shifts in attitudes or qualities, such as empathy, sensitivity, and moral awareness; or it may reflect a sexist or homophobic cultural environment where boys may be particularly prone to expressing acceptance of tolerance of violence against women in general and IPVAW in particular, to be accepted by their peers.

Regarding education, some studies show that various attitudes accepting IPVAW, such as victim blaming, are common among less educated respondents (Gracia and Tomás, 2014). In this sense, Wang’s revision (2016) concluded that “education might be the most crucial among all the factors (associated with attitudes concerning IPVAW), since factors such as age, gender and residency substantively reflected different education levels, and factors such as economic status (…) were deeply rooted in education” (p. 72). Specifically, the research studies were carried out in low-income countries and pointed out that: in general, a lower level of education was positively connected with justifying IPVAW, while higher levels of education decreased the risk of justifying this violence; women who lived in rural areas or without education were more likely to justify IPVAW, while women with secondary or higher levels of education were less likely to accept this violence; and different attitudes between younger and older people can be the result of different education levels—younger age may sometimes be associated with the acceptance of IPVAW because it could represent a lack of experience, knowledge, or education, reflecting an attitude that may change over time with the accumulation of considerable experience. Without further education, however, attitudes toward IPWAW will not improve. While these findings were really interesting and suggestive, they were based on a qualitative revision, not on a systematic search or meta-analysis. Some subsequent empirical studies tend to confirm these results (Sardinha and Najera, 2018).

In summary, as Gracia and Lila (2015) point out after their revision of European surveys and research, “little knowledge or negative attitudes (toward IPVAW) were more common among males, the older, the less educated, and with lower income, minority groups, and those living in rural areas” (p. 110). Regarding the measurement and importance of IPVAW supportive attitudes, “the availability of reliable and valid measures of the acceptability of IPVAW is important for research and intervention purposes” (Gracia et al., 2015, p. 27), with considerable effort made in recent years to develop them.

As noted above, most of research to date about this topic has been conducted through self-reports or explicit assessment measures of attitudes (Eckhardt et al., 2012; Gracia et al., 2015). However, for sensitive areas, such as IPVAW, respondents may avoid sharing their real beliefs because of a fear of negative consequences or judgments and often provide distorted or socially desirable responses, give inaccurate information, or present themselves in a socially acceptable manner (De Houwer et al., 2009; Eckhardt et al., 2012; Gracia et al., 2015; Guan et al., 2017).

Given this probable bias of direct or explicit measures, it has been suggested that they should be complemented by indirect or implicit measures (Fazio and Olson, 2003; Eckhardt et al., 2012). In this sense, some researchers have used implicit methods of assessment that measure attitudes at an indirect level and can provide information that is distinct from self-reports (Olson and Fazio, 2004; Nosek et al., 2011). These implicit measures are strong and widespread on average (Nosek et al., 2007a; Payne et al., 2010), and aggregate scores of these measures at the macro level, such as states or countries, show strong relations with indicators of discrimination at the same level of analysis (Nosek et al., 2009).

As Gawronski (2007) pointed out, indirect attitude measures “differ from traditional self-report measures, in that they do not require explicit evaluations of an attitude object. Rather, attitudes inferred from these measures are based on participants’ performance on experimental paradigms, such as sequential priming or response interference tasks” (p. 573).

Some alternatives have been explored, such as the Implicit Association Test (IAT, Greenwald et al., 1998), one of the best-researched implicit instruments (Gawronski and Payne, 2010). The IAT is an indirect measure used to assess the strength of cognitive associations (attitudes) between two target concepts and an evaluative dimension by utilizing a number of response discrimination tasks and comparing response latency (RL, in milliseconds) to different pairings of the concepts of interest with target stimuli (Greenwald et al., 1998; Fazio and Olson, 2003). The preferences of the individual are inferred from the speed of responding to stimuli in these categorization tasks (Greenwald et al., 2003; Nosek et al., 2007b; De Houwer et al., 2009; Bar-Anan and Nosek, 2014). And this assessment tool has been increasingly used for analysis in several areas of social cognition and social phenomena (Payne and Gawronski, 2010), and it has quickly become the most frequently chosen implicit measurement tool for many relevant social, cognitive, and personality variables (Olson and Fazio, 2004; Nosek et al., 2014).

Particularly, some studies (Robertson and Murachver, 2007; Eckhardt et al., 2012; Eckhardt and Crane, 2014) have used different versions of the IAT to assess attitudes toward women, attitudes toward violence, or attitudes toward the connection between violence and women or gender in men with a history of IPVAW. To our knowledge, however, no previous studies had used the IAT specifically to assess implicit attitudes toward IPVAW, which is why it was necessary to build a personalized IAT form [the Gender Violence Implicit Association Test (GV-IAT), (Ferrer-Perez et al., 2018), as an implicit measure of attitudes toward IPVAW.

The main aim of this study was to examine how implicit and explicit attitudes toward IPVAW are related among a Spanish population. For this general objective, the following specific objectives were set: (1) to estimate the correlation and the convergence–divergence between implicit and explicit measures of attitudes toward IPVAW and (2) to explore the gender and training effect on implicit and explicit measures of attitudes toward IPVAW.

Given the previous knowledge about attitude measurement (Fazio and Olson, 2003; Tosi et al., 2018; Kurdi and Banaji, 2019), a low correlation between implicit and explicit measures was expected. And given the results obtained in the study of attitudes toward IPVAW, previously commented, we hypothesized the following: (i) An effect of education level is expected, in both attitude measures (implicit and explicit measures). Specifically, it is hypothesized that among an educated sample, the levels of rejection will be high. (ii) An effect of gender, participation, and involvement in activities specifically related to IPVAW (formation on this topic) is expected, both in implicit and explicit measures. Specifically, it is hypothesized that women and people who have received previous IPVAW training will show higher levels of rejection and lower levels of acceptability of this violence.

## Materials and Methods

### Participants

An opportunity sample of 693 students from different educational levels (i.e., secondary education, professional training, and undergraduate students) with an average age of 20.03 years (*SD* = 2.34; range: 18–29) took part in this study. The sample characteristics are described in Table 1.

**TABLE 1:** Participant characteristics.

		Total (*n* = 693)
Gender	Men	176(25.4%)
	Women	515(74.3%)
	Not responding	2(0.3%)
Partner	Yes	321(46.3%)
	No	371(53.5%)
	Not responding	1(0.1%)
Educational level	Secondary	59(8.5%)
	Professional training	74(10.7%)
	University studies	559(80.7%)
	Not responding	1(0.1%)
Previous knowledge about IPVAW	Yes	363(52.4%)
	No	328(47.3%)
	Not responding	2(0.3%)
Activities related to IPVAW	Yes	340(49.1%)
	No	353(50.9%)
	Not responding	0(−)
Political opinion	Left	315(45.5%)
	Center	214(30.9%)
	Right	72(10.4%)
	Other (unspecified)	67(9.7%)
	Not responding	25(4.0%)

*IPVAW, intimate partner violence against women.*

### Measures

The *Inventory of Distorted Thoughts about Women and Violence* [IPDMV, the Spanish acronym, Echeburúa and Fernández-Montalvo, 1998; adapted version of Ferrer-Pérez et al. (2019)] comprises 24 items, with a four-point response scale and four dimensions: *Inferiority of Women Compared to Men* (F1-IW, 7 items, α = 0.86; e.g., item 1: Women are inferior to men); *Blaming Female Victims of Abuse* (F2-BW, 7 items, α = 0.62; e.g., item 10: If women really wanted to, they would know how to prevent violence); *Violence as an Appropriate Problem-solving Strategy* (F3-VP, 5 items, α = 0.69; e.g., item 17: Slaps are sometimes necessary); and *Minimization and Exoneration of the Abuser* (F4-MA, 4 items, α = 0.53; e.g., item 29: Men with a history of IPVAW are people with serious psychological problems who often don’t know what they’re doing). Cronbach’s α coefficients for the IPDMV factors in this sample showed slightly lower values than previously reported (F1-IW, α = 0.70; F2-BW, α = 0.54; F3-VP, α = 0.61; and F4-MA, α = 0.45). Higher scores indicate higher levels of distorted thoughts, and given the content of the dimensions analyzed, the lower the values, the stronger the explicit rejection of IPVAW. Cronbach’s α coefficients for the IPDVM factors in this sample showed slightly lower values than previously reported, possibly due to the homogeneity of the sample; however, all of the items contribute to their respective factors’ internal consistency. Despite the relatively low reliability of some of the subscales, reliability values around.50 can be considered acceptable for the purpose of basic research studies (Nunnally, 1967; Schmitt, 1996).

The *Inventory of Beliefs about Intimate Partner Violence* (IBIPV, García-Ael et al., 2018) is a 22-item self-report scale, with a seven-point response scale, designed to assess participants’ explicit attitudes toward IPVAW, grouped into three subscales: *Justifying Partner Violence* (F1-JPV, 6 items, α = 0.71; e.g., item 1: Sometimes men are justified in beating up their partner), *Victims Responsible for Violence* (F2-VRV, 9 items, α = 0.93; e.g., item 7: Battered women are responsible for their abuse, because they intended it to happen), and *Abuser Responsible for Violence* (F3-ARV, 7 items, α = 0.84; e.g., item 16: Abusers are responsible for the abuse because they intended to behave that way from the beginning). Also, Cronbach’s α coefficients for the IBIPV factors in this sample showed slightly lower values than previously reported. In this case, the elimination of items 3, 15, 21, and 22, whose corrected homogeneity indexes were negative or excessively low (< 0.30), improved the reliability, increasing their values in our sample from.318 to.642 for F1-JPV, from.70 to.89 for F2-VRV, and from.82 to.94 for F3-ARV. Additionally, the F3-ARV item scores were recoded so that low scores indicated rejection of violence against women, in the same sense as the other items on the scale. Thus, and in the case of IBIPV, higher scores indicate higher levels of distorted thoughts. Given the content of the dimensions analyzed and the correction made, the lower the values, the stronger the explicit rejection of IPVAW.

The *Gender Violence Implicit Association Test* (GV-IAT, Ferrer-Pérez et al., 2018; Sánchez-Prada et al., 2018) is a form of personalized IAT used as an implicit measure of attitudes toward IPVAW with two target categories, *Gender Violence* vs. *Non-Gender Violence* (the most distinctive feature of this personalized IAT) and two attribute categories, *Good* vs. *Bad*. Regarding the target category, Gender Violence vs. Non-Gender Violence, it is important to note that under Spanish law (Organic Act 1/2004, of 28 December, on Integrated Protection Measures against Gender Violence), IPVAW is known as gender violence (see Ferrer and Bosch, 2014), which is why GV-IAT uses gender violence to refer to IPVAW.

Stimuli were displayed on a 20-inch screen with a PC running OpenSesame v.3.1.6 (Mathôt et al., 2012) on Windows 8.

According to the literature review, the typical IAT procedure with feedback was used (Greenwald et al., 1998; Nosek et al., 2007a, 2014; Sánchez-Prada et al., 2018). Participants completed the GV-IAT task in seven blocks (Greenwald et al., 2003): The first block was the initial target concept discrimination task, which included 24 practice trials, where participants sorted non-gender-violence (i.e., tolerate, support, respect, empathize, cooperate, and collaborate) and gender-violence (i.e., torture, infringe, humiliate, hit, force, and attack) behaviors into the categories “non-gender-violence” and “gender-violence” using the “s” key (left) and the “l” key (right). The second block involved an associated attribute concept discrimination task, which included 24 practice trials, and participants sorted words into “bad” (i.e., worst, terrible, negative, horrible, disastrous, and appalling) and “good” (i.e., wonderful, positive, phenomenal, optimum, excellent, and best) categories. The third block was a congruent combination task in which words corresponding to both the target concept (non-gender-violence/gender-violence) and the attribute concept (good/bad) flashed on the computer screen (non-gender-violence/good, gender-violence/bad), and it included 24 critical trials. The fourth block was in the same form as the third block but included 48 critical trials. The fifth block included 48 practice trials, and the reversed target concept discrimination task was presented so that participants sorted non-gender-violence (e.g., respect) and gender-violence (e.g., humiliate) behaviors into the categories “non-gender-violence” and “gender-violence” using the “s” key (right) and the “l” key (left). The sixth block was an incongruent combination task in which words corresponding to both the target concept (non-gender-violence/gender-violence) and the attribute concept (good/bad) flashed on the computer screen (non-gender-violence/bad, gender-violence/good), and it included 24 critical trials. And the seventh block was in the same form as the sixth block but included 48 critical trials.

Within all blocks, words were presented in random order; one-half of the participants completed the GV-IAT described, and the other half, a GV-IAT counterbalanced (half to the left, half to the right) in the order of congruent and incongruent blocks (so the order of the blocks was B1, B2, B6, B7, B5, B3, and B4), to reduce order effects (Nosek et al., 2005).

The fundamental principle of the IAT is that when two concepts are strongly associated, the RL is less than when this is not the case. The IAT scores are calculated using a latency-based response obtained in the trials corresponding to the compatible critical phase (RLc) for congruent category pairs (e.g., gender-violence/bad) and those obtained in the trials corresponding to the incompatible critical phase (RLi) for incongruent category pairs (e.g., gender-violence/good).

Based on response latencies, and to analyze the GV-IAT effect, a D-score was calculated for each participant, according to the algorithm proposed by Greenwald et al. (2003) and subsequently optimized in different research (Fazio and Olson, 2003; Nosek et al., 2007a; Glashouwer et al., 2013; Nosek et al., 2014; Blanton et al., 2015), as we have described previously as another feature of this personalized IAT (Ferret et al., in press). For the error latencies’ treatment, we used the procedure “built-in penalty” in error latencies by computing the accumulated time of the wrong-plus-corrected response (Greenwald et al., 2003; Nosek et al., 2014; Richetin et al., 2015).

D-scores obtained were interpreted similarly to Cohen’s d (Cohen, 1988), in the same sense proposed by Greenwald et al. (2003). Thus, in GV-IAT, positive D-scores express a longer latency time when gender violence is associated with positive stimuli (e.g., gender-violence/good) than when it is associated with negative stimuli (e.g., gender-violence/bad). This indicates a perception of incongruence between positive stimuli and gender violence (i.e., RLi greater than RLc) or implicit rejection of gender violence: the higher the value of D, the stronger the rejection. In turn, values close to zero or negative D-scores express low differences in latency times, which indicate a perception of congruence between positive stimuli and gender violence (i.e., RLi similar or less than RLc) or an implicit acceptance of gender violence. In summary, positive D-scores in GV-IAT indicate implicit rejection of IPVAW, and the higher the values, the stronger the rejection.

Finally, participants completed a brief questionnaire designed *ad hoc*, including the following information: some sociodemographic data (gender, age, educational level, previous IPVAW training, and political opinion); a feeling *thermometer* (Greenwald and Farnham, 2000; Greenwald et al., 2003) composed of a question (“Among the various, and sometimes contradictory feelings that social problems can provoke, to what extent do women who claim to be IPVAW victims elicit this feeling in you?”) that participants responded to twice, once about positive feelings (on a 10-point response scale from indifference to solidarity and tenderness) and once about negative feelings (on a 10-point response scale from indifference to irritation); and some questions about the perceived severity (from 1 = nothing serious to 4 = very serious) of different forms of IPVAW (physical violence, psychological violence, sexual violence). When asking about IPVAW perceived severity, we used exactly the same questions used in the 2010 Eurobarometer (European Commission, 2010) for this aim.

### Procedure

The recruitment process was carried out by announcing the study in high schools, professional training centers, and the universities where the authors teach and by requesting volunteers who did not receive any incentive for their participation.

The study was approved by the Ethics Research Committee (University of Balearic Islands, Spain), and experimental sessions took place in the labs at each university. Upon arrival, participants were asked to read over the study description and written consent form. Also, the study materials and consent forms were developed in accordance with ethical norms and guidelines from all participating countries and universities. To properly meet these standards, it was necessary to include in the informed consent what the purpose of the study was (to study attitudes toward IPVAW).

After providing their informed consent to voluntary participation in the study, the participants completed the IAT and, thereafter, the two questionnaires of explicit measures, as well as the brief questionnaire designed *ad hoc*.

## Results

### Implicit Measures

#### Internal Consistency and Construct Validity Evidence

In order to use algorithm D with the greatest effect on any of the psychometric criteria (Nosek et al., 2014), D-scores were estimated in two ways: (a) computing all trials in each block (B1–B2 included 24 trials; B5 included 48 trials) and (b) removing the first warm-up trials (B1–B2 included 20 trials; B5 included 40 trials). The internal consistency (split-half reliability; Greenwald, et al. 2003) was estimated following the procedure recommended by Kurdi et al. (2019) through an online tool (available at ^1^). The distribution of the 600 split-half correlations calculated by this procedure yielded an average estimate of.73 for both the two D-scores’ estimation ways, after applying a Spearman–Brown correction (De Houwer and De Bruycker, 2007; Carpenter et al., 2019). This value is within the range of.70 to.90 (split-half correlations or alphas) considered acceptable for the IAT measures (Schmukle and Egloff, 2004; Nosek et al., 2007a).

The available examples of construct validity were explored using two types of evidence based on the relationship with other variables (AERA et al., 2014): the correlation with feeling *thermometer* items (Greenwald and Farnham, 2000) and the independent variable’s influences on the IAT (Nosek et al., 2007a). Correlations between the GV-IAT scores and the feeling *thermometer* items were low, but when D-scores were estimated by removing the first warm-up trials, there was a positive statistically significant correlation with feelings of solidarity toward the victims (*r* = 0.08; *p* = 0.04) and a negative statistically significant correlation with feelings of irritability (*r* = -0.09; *p* = 0.03). Similarly, correlations between the GV-IAT scores and the perceived severity of IPVAW were low, but when D-scores were estimated by removing the first warm-up trials, there was a positive statistically significant correlation with perceived severity of physical (*r* = 0.13; *p* = 0.001), psychological (*r* = 0.08; *p* = 0.04), and sexual abuse (*r* = 0.08; *p* = 0.03).

#### Implicit Explicit Measures’ Correlation

Correlations between GV-IAT and explicit measures of attitudes toward IPVAW were low, in line with what has been reported in the literature (Hofmann et al., 2005; Gawronski and Payne, 2010; Tosi et al., 2018; Kurdi et al., 2019), with the results being slightly higher when D-scores were estimated by removing the first warm-up trials (see Tables 2, 3). It is also worth mentioning that the correlations were negative in all cases, which, given the inverse meaning of the scores on both types of measures (i.e., the higher the values, the stronger the rejection in the case of implicit measures, and the lower the values, the stronger the rejection in the case of explicit measures), would mean that those who showed rejection on implicit measures also showed it on explicit ones, with the same occurring for acceptance.

**TABLE 2:** Correlations between implicit and explicit measures — GV-IAT–IPDVM scores.

	F1 IW (*n* = 692)	F2 BW (*n* = 675)	F3 VP (*n* = 686)	F4 MA (*n* = 679)	Total IPDVM (*n* = 659)
GV-IAT 24–48 trials	*r*_xy_ = −0.138 *p* < 0.001	*r*_xy_ = −0.050 *p* = 0.193	*r*_xy_ = −0.031 *p* = 0.425	*r*_xy_ = −0.013 *p* = 0.736	*r*_xy_ = −0.026 *p* = 0.501
GV-IAT 20–40 trials	*r*_xy_ = −0.188 *p* < 0.001	*r*_xy_ = −0.106 *p* = 0.006	*r*_xy_ = −0.062 *p* = 0.107	*r*_xy_ = −0.042 *p* = 0.277	*r*_xy_ = −0.073 *p* = 0.062

*Negative correlation indicates positive association between implicit and explicit rejection. GV-IAT, Gender Violence Implicit Association Test; IPDMV, Inventory of Distorted Thoughts about Women and Violence; F1-IW, Inferiority of Women Compared to Men; F2-BW, Blaming Female Victims of Abuse; F3-VP, Violence as an Appropriate Problem-solving Strategy; F4-MA, Minimization and Exoneration of the Abuser.

**TABLE 3:** Correlations between implicit and explicit measures—GV- IAT–IBIPV scores.

Rejection level	F1 JPV (*n* = 597)	F2 VRV (*n* = 602)	F3 ARV (*n* = 595)	Total IBIPV (*n* = 579)
GV-IAT 24–48 trials	*r*_xy_ = −0.050 *p* = 0.124	*r*_xy_ = −0.145 *p* < 0.001	*r*_xy_ = −0.105 *p* = 0.011	*r*_xy_ = −0.062 *p* = 0.139
GV-IAT 20–40 trials	*r*_xy_ = −0.072 *p* = 0.078	*r*_xy_ = −0.158 *p* < 0.001	*r*_xy_ = −0.138 *p* < 0.001	*r*_xy_ = −0.078 *p* = 0.061

*Negative correlation indicates positive association between implicit and explicit rejection. IBIPV, Inventory of Beliefs about Intimate Partner Violence; F1-JPV, Justifying Partner Violence; F2-VRV, Victims Responsible for Violence; F3-ARV, Abuser Responsible for Violence.

#### Differences in Implicit Measures by Sociodemographic and Related IPVAW Variables

Gender Violence Implicit Association Test score comparisons by some variables related with IPVAW attitude were made using ANOVA (Table 4). Previously, a combined variable was created *ad hoc* from previous knowledge about IPVAW (Yes/No) and participation in activities on this topic (Yes/No), thus establishing two groups of subjects: those involved with IPVAW (Yes knowledge + Yes activities) and those not involved (No knowledge + No activities).

**TABLE 4:** GV-IAT mean score comparisons.

Variable		M	SD	Effect	Effect size
Gender	Men (*n* = 176)	0.64	0.29	*F* (1, 689) = 6.492 *p* = 0.011	η^2^ = 0.009
	Women (*n* = 515)	0.70	0.29		
Partner	No (*n* = 371)	0.70	0.28	*F* (1, 689) = 0.787 *p* = 0.375	--------
	Yes (*n* = 321)	0.68	0.29		
IPVAW knowledge	No (*n* = 328)	0.66	0.29	*F* (1, 689) = 4.595 *p* = 0.032	η^2^ = 0.007
	Yes (*n* = 363)	0.72	0.29		
IPVAW activities	No (*n* = 353)	0.67	0.28	*F* (1, 689) = 2.930 *p* = 0.087	--------
	Yes (*n* = 340)	0.71	0.30		
IPVAW involvement	No (*n* = 196)	0.64	0.29	*F* (1, 402) = 5,736 *p* = 0.017	η^2^ = 0.014
	Yes (*n* = 208)	0.71	0.31		
Political opinion	Right (*n* = 72)	0.67	0.32	*F* (3, 660) = 2.656 *p* = 0.048	η^2^ = 0.012
	Center (*n* = 214)	0.69	0.28		
	Left (*n* = 315)	0.71	0.28		
	Other (*n* = 67)	0.63	0.34		

These results show that, although the effect sizes are small, the D-scores are significantly higher among women with prior knowledge of IPVAW and, with a larger effect size, among those having greater involvement with IPVAW. In terms of political opinion, there is greater rejection among left-wing subjects than right-wing subjects and other (unspecified) political options.

**FIGURE 1:**
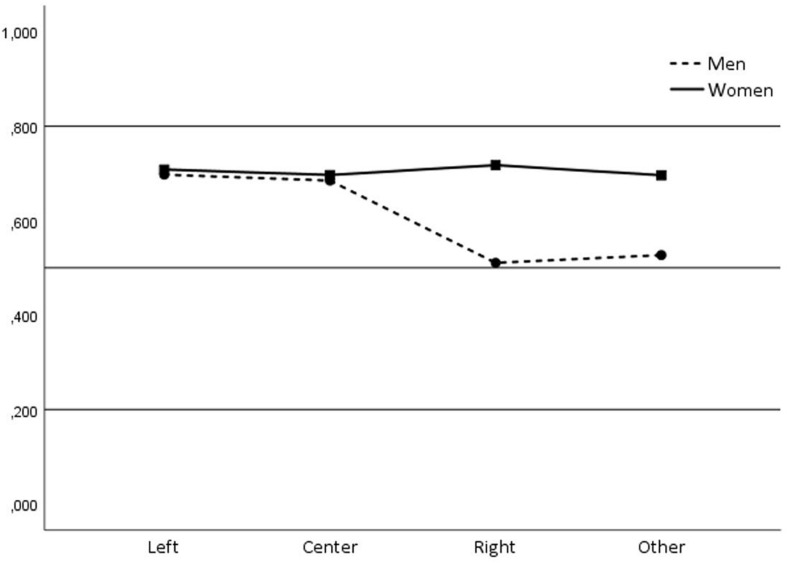
Interaction effect of gender * political opinion.

In terms of interaction, only significant effects of the interaction between gender and political opinion were observed [*F* (3, 660) = 2.790, *p* = 0.040, η^2^ = 0.013], so there is no difference between men and women who identified with left-wing and center political leanings in their implicit rejection of IPVAW. However, men who identified with right-wing and other political options show less implicit rejection toward IPVAW than women (Figure 1).

### Explicit Measures

#### Comparison Between Explicit Measures’ Factors and Interaction With Gender

To compare the different factors of attitudes toward IPVAW by gender, two repeated measures mixed ANOVAS were performed, taking gender as an inter-subject variable and factor scores as an intra-subject variable. Given the different metrics of the instruments (scale from 1 to 4 for IPDMV and from 1 to 7 for IBIPV), two different analyses were performed, one for each instrument.

**FIGURE 2:**
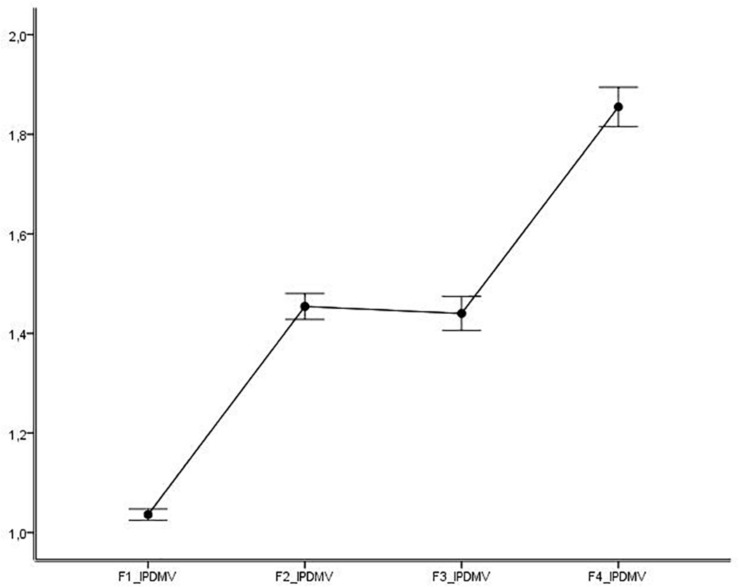
IPDMV factors’ comparison (95% confidence intervals). IPDMV, Inventory of Distorted Thoughts about Women and Violence; F1-IW, Inferiority of Women Compared to Men; F2-BW, Blaming Female Victims of Abuse; F3-VP, Violence as an Appropriate Problem-solving Strategy; F4-MA, Minimization and Exoneration of the Abuser.

The comparison between the factors of the IPDMV, with Greenhouse–Geisser non-sphericity correction epsilon of freedom degrees, was significant [*F* (2.388, 1,563.857) = 496.924, *p* < 0.001, η^2^ = 0.431), with significant differences in all pair-wise comparisons (*p* < 0.001), except between F2-BW and F3-VP (*p* = 1.0). Figure 2 shows this effect.

**FIGURE 3:**
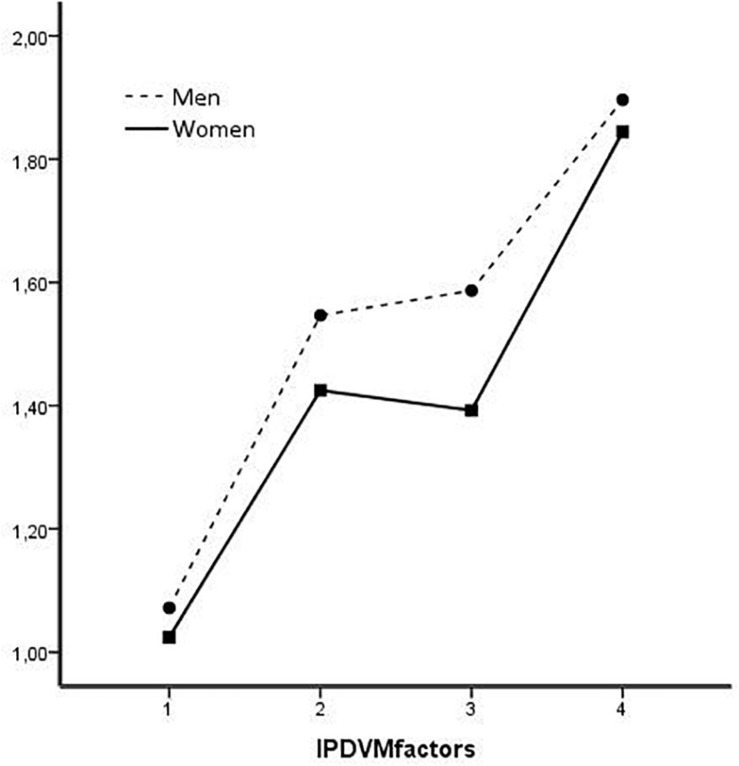
Gender * IPDMV factors interaction.

The interaction gender ^∗^ IPDMV factors was also significant [*F* (2.388, 1,563.857) = 5.272, *p* = 0.003, η^2^ = 0.008] indicating that differences between men and women vary depending on the factor. This interaction effect can be seen in Figure 3, so the difference by gender is greater for F2-BW and F3-VP and smaller for F1-IW and F4-MA.

**FIGURE 4:**
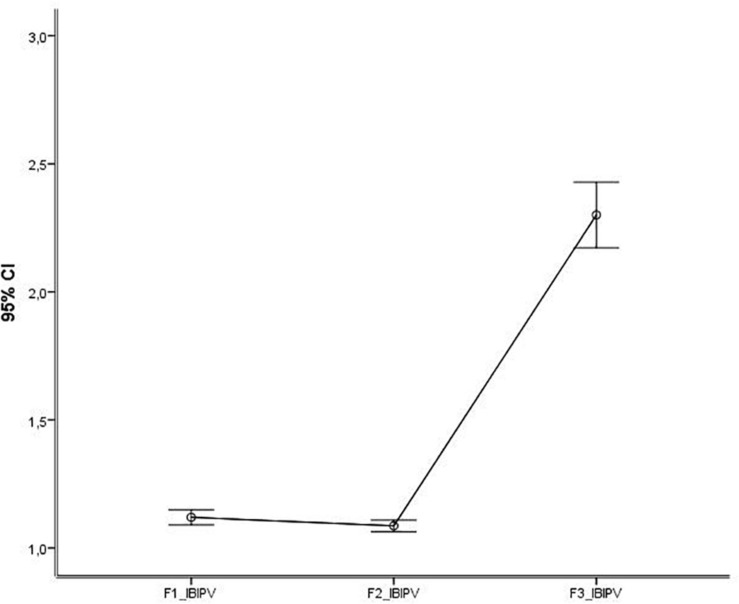
IBIPV factors’ comparison (95% confidence intervals). IBIPV, Inventory of Beliefs about Intimate Partner Violence; F1-JPV, Justifying Partner Violence; F2-VRV, Victims Responsible for Violence: F3-ARV, Abuser Responsible for Violence.

The comparison between the factors of the IBIPV, with Greenhouse–Geisser non-sphericity correction epsilon of freedom degrees, was significant [*F* (1.067, 613.615) = 339.314, *p* < 0.001, η^2^ = 0.371], with differences between F1-JPV and F3-ARV (*p* < 0.001) and F2-VRV and F3-ARV (*p* < 0.001) but not between F1-JPV and F2-VRV (*p* = 0.061). Figure 4 shows this effect.

**FIGURE 5:**
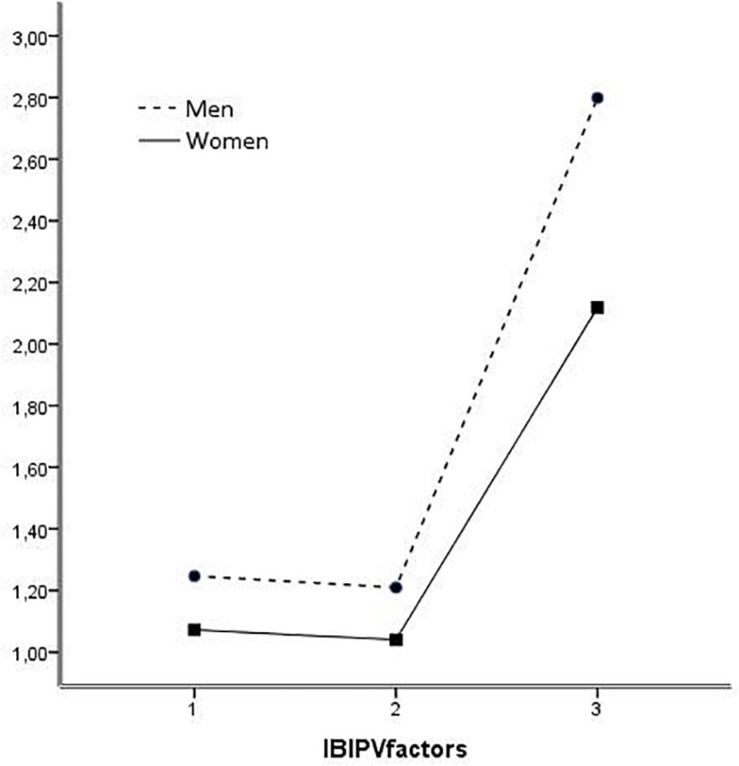
Gender * IBIPV factors interaction.

The interaction gender ^∗^ IBIPV factors was also significant [*F* (1.067, 613.615) = 12.628, *p* < 0.001, η^2^ = 0.021]. Figure 5 shows this effect and the increase in the differences by gender in F3-ARV, relating to F1-JPV and F2-VRV.

#### Differences in Explicit Measures by Sociodemographic and Related IPVAW Variables

The effects of having a partner, involvement in IPVAW, and political opinion, as well as the interaction of these variables with gender, were analyzed using MANOVA (Pillai’s Trace). No significant effects were obtained for either having a partner [*F* (7, 444) = 0.713, *p* = 0.661] or IPVAW involvement [*F* (14, 890) = 1.395, *p* = 0.149]. There were also no interaction effects for gender ^∗^ having a partner [*F* (7, 444) = 1.954, *p* = 0.060] or for gender ^∗^ IPVAW involvement [*F* (14, 890) = 1,014, *p* = 0.436]. Significant effects were only obtained for political opinion [*F* (21, 1,455) = 5.069, *p* < 0.001, η^2^ = 0.068], although without interaction effects for political opinion ^∗^ gender [*F* (21, 1,451) = 1.137, *p* = 0.301]. In all factors, the sense of the differences between different political ideas is the same, regardless of the gender of the subjects, and in all factors, the acceptance scores are higher for men than for women of the same political ideology, although in F1-IW and F3-VP, the differences between men and women are not statistically significant. The univariate contrasts of political opinion on the various factors of explicit measures are presented in Table 5, with F3-VP having the largest effect size (12.7%).

**TABLE 5:** Differences in explicit measures by political opinion.

Factor		*M*	*SD*	Effect	Effect size
IPDMV F1-IW	Left (*n* = 261)	1.01	0.09	*F* (3, 528) = 5.42 *p* < 0.001	η^2^ = 0.030
	Center (*n* = 173)	1.04	0.13		
	Right (*n* = 52)	1.06	0.20		
	Other (*n* = 50)	1.10	0.29		
IPDMV F2-BW	Left (*n* = 261)	1.34	0.27	*F* (3, 528) = 17.45 *p* < 0.001	η^2^ = 0.090
	Center (*n* = 173)	1.52	0.35		
	Right (*n* = 52)	1.55	0.35		
	Other (*n* = 50)	1.57	0.43		
IPDMV F3-VP	Left (*n* = 261)	1.29	0.33	*F* (3, 528) = 25.49 *p* < 0.001	η^2^ = 0.127
	Center (*n* = 173)	1.53	0.45		
	Right (*n* = 52)	1.83	0.50		
	Other (*n* = 50)	1.52	0.58		
IPDMV F4-MA	Left (*n* = 261)	1.73	0.48	*F* (3, 528) = 7.28 *p* = 0.048	η^2^ = 0.040
	Center (n = 173)	1.90	0.46		
	Right (*n* = 52)	2.05	0.51		
	Other (*n* = 50)	1.85	0.63		
IBIPV F1-JPV	Left (*n* = 261)	1.08	0.28	*F* (3, 528) = 2.43 *p* = 0.065	*———–*
	Center (*n* = 173)	1.12	0.37		
	Right (*n* = 52)	1.16	0.41		
	Other (*n* = 50)	1.23	0.61		
IBIPV F2-VRV	Left (*n* = 261)	1.04	0.12	*F* (3, 660) = 11.86 *p* < 0.001	η^2^ = 0.063
	Center (*n* = 173)	1.09	0.31		
	Right (*n* = 52)	1.13	0.28		
	Other (*n* = 50)	1.25	0.55		
IBIPV F3-ARV	Left (*n* = 261)	2.05	1.40	*F* (3, 528) = 4.88 *p* = 0.002	η^2^ = 0.027
	Center (*n* = 173)	2.19	1.48		
	Right (*n* = 52)	2.58	1.76		
	Other (*n* = 50)	3.04	1.69		

**FIGURE 6:**
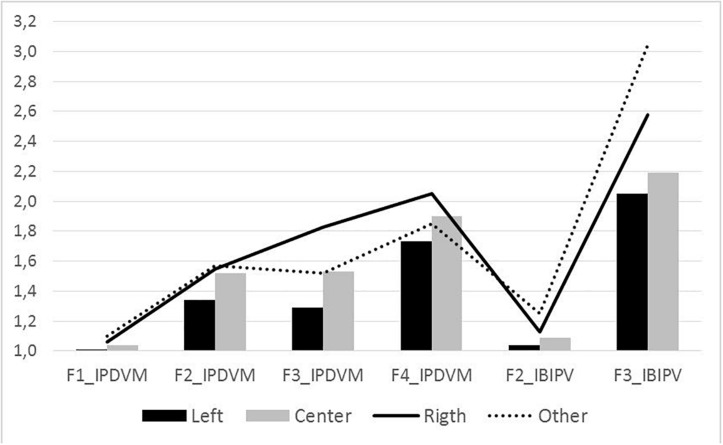
Effects of political opinion in explicit measures.

Figure 6 shows these scores for factors where significant effects of political opinion are observed. Although acceptance of IPVAW is generally low, the table indicates that it is always lower for people identified as left-wing and higher for people identified as right-wing or other options, depending on the factor.

### Explicit vs. Implicit Levels of Rejection

#### Level of Implicit Rejection

In order to classify participants by their implicit rejection of IPVAW, and considering that the higher the D-scores on the GV-IAT, the stronger the rejection, participants were classified into four levels of rejection, according to D-intervals (small, medium, and large IAT effects) and parallel Cohen (1988) values of.2,0.5, and.8, respectively (Greenwald et al., 2003; Eckhardt and Crane, 2014). Table 6 shows the crosstab between participants’ distribution according to these categories and their gender.

**TABLE 6:** Crosstab of GV-IAT D-score classification and gender.

Rejection level	Men (*n* = 176)	Women (*n* = 515)	Total (*n* = 691)
Null (D < 0.2)	20 (11.4%)	36(7.0%)	56(8.1%)
Mild (0.2 ≤ D < 0.5)	28 (15.9%)	70(13.6%)	98(14.1%)
Moderate (0.5 ≤ D < 0.8)	73 (41.5%)	196(38.0%)	269(38.8%)
Strong (D ≥ 0.8)	55 (31.2%)	213(41.4%)	268(38.7%)

The chi-square test shows no significant relationship between gender and the participants’ classification by implicit measures [χ^2^ (3) = 7.442; *p* = 0.059].

#### Level of Explicit Rejection

In order to classify participants by their explicit rejection of IPVAW, following the Likert response scale in each questionnaire, four categories were established: *disagreement* (IPDVM scores ≤ 2 and IBIPV scores ≤ 3) was categorized as *strong rejection*; *near-disagreement* (IPDVM scores between 2 and 2.5 and IBIPV scores between 3 and 4) as moderate rejection; *near-agreement* (IPDVM scores between 2.5 and 3 and IBIPV scores between 4 and 5) as *mild rejection*; and *agreement* (IPDVM scores ≥ 3 and IBIPV scores ≥ 5) as *null rejection*. Tables 7, 8 show the participants’ distribution according to these categories.

**TABLE 7:** Frequency distribution of explicit IPDVM scores.

Rejection level	F1 IW (*n* = 692)	F2 BW (*n* = 675)	F3 VP (*n* = 686)	F4 MA (*n* = 679)	Total IPDVM (*n* = 659)
Null rejection (x ≥ 3)	2(0.1%)	1(0.1%)	7(1.0%)	23(3.3%)	0(−)
Mild rejection (2.5 ≤ x < 3)	0(−)	8(1.2%)	11(1.6%)	51(7.4%)	5(0.7%)
Moderate rejection (2 < x < 2.5)	2(0.3%)	29(4.2%)	44(6.4%)	130(18.8%)	25(3.6%)
Strong rejection (x ≤ 2)	688(99.3%)	637(91.9%)	624(90.0%)	475(68.5%)	629(90.8%)
Not complete Cases	2(0.3%)	18(2.6%)	7(1.0%)	14(2.0%)	34(4.9%)

**TABLE 8:** Frequency distribution of explicit IBIPV scores.

Rejection level	F1 JPV (*n* = 597)	F2 VRV (*n* = 602)	F3 ARV (*n* = 595)	Total IBIPV (*n* = 579)
Null rejection (x ≥ 5)	0(0.9%)	0(−)	52(7.5%)	0(−)
Mild rejection (4 ≤ x < 5)	1(0.1%)	2(0.3%)	15(2.2%)	1(0.1%)
Moderate rejection (3 < x < 4)	3(0.4%)	0(−)	69(9.1%)	5(0.7%)
Strong rejection (x ≤ 3)	593(85.6%)	600(86.6%)	459(66.2%)	573(82.7%)
Not complete cases	96(13.9%)	91(13.1%)	104(15.0%)	114(16.5%)

In general, the explicit measures applied yielded high percentages of strong explicit rejection of IPVAW, particularly in the case of beliefs related to the inferiority of women compared to men, the responsibility of women for the violence experienced, the justification of partner violence, or the consideration of violence as an appropriate problem-solving strategy. But these percentages of strong rejection are lower in the case of the responsibility of men for the violence perpetrated (68.5% in IPDMV F4-MA and 66.2% in IBIPV F3-ARV).

Related to the distribution of explicit rejection levels in each factor by gender, in IPDMV, significant effects were observed for F2-BW [χ^2^ (3) = 21.143; *p* < 0.001] and for F3-VP [χ^2^ (3) = 28.177; *p* < 0.001]. In both cases, women are ranked higher than men in the strong rejection category, and men higher than women in the moderate rejection category. In IBIPV, significant effects are observed for F2-VRV [χ^2^ (1) = 5.290; *p* = 0.021], and F3-ARV [χ^2^ (2) = 17.614; *p* = 0.001], with a higher proportion of women than men in the strong rejection category.

#### Levels of Implicit vs. Explicit Rejection of IPVAW

**FIGURE 7:**
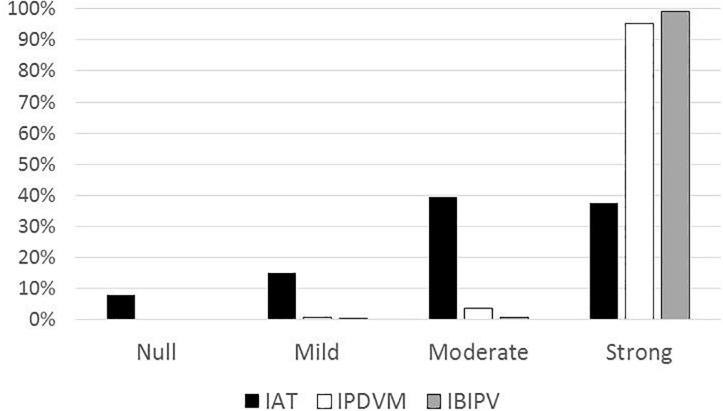
Implicit and explicit (Total-IPDVM and Total-IBIPV) rejection frequency distribution.

Finally, we present the comparison between the participants’ classification by implicit and explicit measures (Figure 7). The distribution obtained describes a sample with strong explicit rejection of IPVAW (over 95% in IPDMV and IBIPV), while the implicit measures (GV-IAT) reflect that less than 40% of the sample reach this strong rejection. Consequently, the chi-square test shows no significant relationship between the participants’ classification by implicit measures and their classification by explicit measures [χ^2^ (6) = 5.845; *p* = 0.441].

## Discussion

In light of the results obtained, we can draw several conclusions. Firstly, these results point out that the psychometric characteristics of the implicit measure used (GV-IAT) are acceptable. Thus, its internal consistency is within a range considered acceptable for the IAT measures (Schmukle and Egloff, 2004; Nosek et al., 2007a). Additionally, its correlation with the feeling thermometer items (Greenwald and Farnham, 2000) and with an independent variable influence on the IAT (Nosek et al., 2007a), such as the perceived severity of IPVAW, may be considered evidence of its construct validity. Moreover, as expected, correlations were low (Hofmann et al., 2005; Gawronski and Payne, 2010; Tosi et al., 2018; Kurdi et al., 2019) between the implicit and explicit measures of attitudes toward IPVAW used (the GV-IAT and the IPDMV and IBIPV self-reports). On the other hand, the sense of the correlations (negative in all cases) indicated that participants who showed rejection on implicit measures also showed it on explicit ones, and participants who showed acceptance on implicit measures also showed it on explicit ones, which could be considered as an indicator that both measures evaluate the same construct (Nosek et al., 2014) or that, at minimum, there may be different aspects of the same construct when the *motivation* to control the explicit responses is expected to be high (Fazio and Olson, 2014), as the Motivation and Opportunity as Determinants (MODE) model hypothesizes. It is worth to noting that the MODE model (proposed by Fazio in 1990) is considered as a useful framework for understanding when attitude and norm accessibility should play an important role in social behavior.

Another interesting topic from a methodological point of view is that correlations between attitudes measured implicitly and the feeling thermometer items, or the perceived severity of IPVAW, increased when D-scores were estimated by removing the first warm-up trials. This result is another example of the particular relevance of the algorithm to calculate the D-score. Unlike Greenwald et al. (2003), in our case, eliminating the first warm-up trials does not change the internal consistency and instead improves the correlation with external criteria, which can be considered as evidence of validity (Greenwald and Farnham, 2000; Nosek et al., 2007a).

With regard to the differences among sociodemographic and IPVAW-related variables, our results point to some important issues. Thus, although the effect sizes were small, as hypothesized, we obtained significant differences by gender, IPVAW knowledge, IPVAW involvement, and political opinion when we measured IPVAW attitudes by implicit measures; however, when we measured these attitudes by explicit measures, we only obtained significant differences by gender and political opinion. By gender, in both implicit and explicit measures, women show more rejection of IPVAW than men. In the case of explicit measures, women are particularly reluctant to blame the victims for the violence they suffer (F2-BW) and to consider violence as an appropriate form of problem solving (F3-VP), while men are reluctant to hold the batterer as responsible for IPVAW (F3-ARV). These results confirm the gender gap in attitudes toward IPVAW described in previous research (Flood and Pease, 2009; Gracia et al., 2015; Herrero et al., 2017; Martín-Fernández et al., 2018). By previous knowledge, we obtained significant differences only in implicit measures, so people with more knowledge and involvement with IPVAW show greater levels of rejection to it. In this case, these results also confirm previous findings (Wang, 2016; Ferrer-Pérez et al., 2019). By political opinion, unlike previous studies (e.g., Delgado and Gutiérrez, 2013), both implicit and explicit measures produce significant differences and similar results. Thus, people with left-wing political tendencies show more IPVAW rejection than those with right-wing tendencies. An interesting phenomenon is that greater acceptance levels of IPVAW occur among people who identified with right-wing and other political options. Specifically, men who identified with these political leanings show less implicit rejection of IPVAW, while both men and women who identified with these political ideas show greater levels of IPVAW acceptance for all factors explicitly measured, except in the case of justifying partner violence (IBIPV F1-JPV). The greatest differences were for the acceptance of violence as an appropriate problem-solving strategy (IPMDV F3-VP) and the two factors that measure batterer absence of responsibility in IPVAW (IPDMV F4-MA and IBIPV F3-ARV). Although these results need to be studied in depth, we hypothesize that they could be related to the emergence of extreme right-wing groups in the Spanish political scene in the last few years.

Finally, regarding the levels of IPVAW rejection, the results highlight the important differences between the levels of strong IPVAW rejection measured with explicit and implicit measures (91–83% when we use self-reports measures, 39% when we use GV-IAT). These results therefore confirm the traditional discrepancy between explicit and implicit measures of attitudes (Fazio and Olson, 2003), and the low correlations among them, from an average of.24 [90% CI = 0.01–0.47], found in the meta-analysis of Hofmann et al. (2005), to the r = 0.12 estimate [90% CI = -0.12–0.33] obtained by Kurdi and Banaji (2019), adding further arguments to the discussion about the problems associated with the attitudes’ measurement (Fazio and Olson, 2014). In fact, correlations between implicit and explicit measures tend to be so low that some authors question whether this is a convergent or a discriminant validity criterion (Tosi et al., 2018), and the debate is still open as to how the results should be interpreted (Fazio and Olson, 2014). One of the available models to explain these results may be the Associative–Propositional Evaluation (APE) model (Gawronski and Bodenhausen, 2006), a dual-process theory that specifies the relation between explicit and implicit evaluations, assuming that they are the product of two functionally distinct mental processes: spontaneous implicit evaluations would be the outcome of associative processes, and deliberate explicit evaluations would be the outcome of propositional processes.

Another interesting point is that, from the point of view of explicit measures, this sample indicates high rejection of the IPVAW, which could be simply related to its composition (i.e., women, highly educated), as indicated by the literature on the subject (Wang, 2016). However, as can we see, not all aspects of IPVAW generate the same level of rejection, with rejection being much higher in relation to blaming the victim and much lower when it comes to the responsibility of the perpetrator. In fact, given that in both explicit measures (IPDMV and IBIPV), the aspect of IPVAW that produces the highest level of acceptance is precisely the (non)responsibility of men with a history of IPVAW, this is a very important aspect to take into account for future research, and possible interventions, with similar populations.

Additionally, we discovered a significant relationship between gender and the IPVAW rejection levels’ classification for explicit measures but not for implicit measures. Thus, by explicit measures, women are ranked higher than men in the strong rejection category for the factors “blaming female victims of abuse,” “violence as an appropriate problem-solving strategy,” “victims as responsible for violence,” and “abuser (as not) responsible for violence.” It may be worth mentioning that, in the case of implicit measures, there is not a significant relationship, but there is a tendency to a different rejection level. In fact, if we compare women and men with null and strong IPVAW implicit rejection, the percentage of women who show rejection is higher. Again, these results confirm the gender gap in acceptance of IPVAW, previously commented (Flood and Pease, 2009; Gracia et al., 2015; Herrero et al., 2017; Martín-Fernández et al., 2018).

### Strengths and Limitations

Although our study has a number of strengths, it also comes with a number of limitations and challenges. The main strength is that it is one of the few works to evaluate the implicit attitudes toward IPVAW through the IAT (in this case applying a personalized form of the IAT, the GV-IAT). Additionally, the results obtained and previously described in this study may lead to the design of preventive actions and public policies on IPVAW and to the use of this assessment tool as a complementary method using the shared variance between IAT scores and explicit measures to control for measurement error in both methods, incorporating a multimethod approach into the measurement of attitudes toward IPVAW (Schimmack, 2019) both in the general population and in specific ones (such as men with a history of IPVAW).

Despite its strengths, this proposal also comes with limitations and challenges. To begin, the main limitation is probably the high homogeneity of the sample studied, made up of a large number of young female undergraduates, who commonly show (as in this case) a high rate of IPVAW rejection (Wang, 2016). This homogeneity might have affected not only the correlations between implicit and explicit measures (Kurdi and Banaji, 2019) but also the possibility of finding clearer differences in the comparisons performed as well. It is therefore necessary to contrast our results in broader and more heterogeneous samples and, particularly, with a more balanced sample size across sex to obtain more consistent conclusions.

Additionally, the nature of the IAT itself and, consequently, the characteristics of its personalized forms, such as the GV-IAT, might imply some limitations. For example: the influence of cognitive inertia or lack of cognitive capability may be a relevant issue with some samples (Messner and Vosgerau, 2010; Romero-Martínez et al., 2013; Gracia et al., 2015); indirect or implicit measures, although less dependent on language and more resistant to participant manipulation, are not impervious to impression management or simple non-compliance (Eckhardt et al., 2012); as with all implicit measures, there is a lack of direct procedures to obtain conclusive evidence on the validity of the construct, given the involvement of different sources of variation in the measurements obtained; and the discrepancy between indirect or implicit and direct or explicit measures is more the standard than the exception (Hofmann et al., 2005; Kurdi and Banaji, 2019). In summary, the processes involved in IAT measures are complex, and the fundamental debate on the validity of implicit measures in general is still open (Gawronski and Hahn, 2019). Additionally, there are different indirect or implicit methods of measuring attitudes (e.g., the PVAMT, Gracia et al., 2015), whereas only the GV-IAT was used in this study.

The relatively low internal consistency of the explicit measures could also be considered as a limitation. In this sense, it should be noted that the assessment of a reliability estimator, like any other evidence of validity, is closely related to the use that will be made of the measure (AERA et al., 2014), and as Hogan (2003) points out, “it depends on what you want to do with the test” (p. 163). In summary, “Test reliability may be crucial in some settings (e.g., those in which major decisions are made on the basis of tests), but less important in others (e.g., where tests are used only for preliminary screenings)” (Nunnally and Bernstein, 1994, p.142). And considering that the estimation of reliability in the General Linear Model depends on the variability of the sample and the length of the instrument, any value must be contextualized (Muñiz, 2018). Therefore, it is worth mentioning the low number of items in some dimensions of the questionnaires used, taking into account that, in general, “reliability of item clusters, say three or four items, is notoriously low, at best usually around.30–0.40” (Hogan, 2003, p. 156). Similarly, it seems necessary to point out the homogeneity of the sample studied, since “if a sample selected on some narrow criterion is used, such as students, then, reliability coefficients will be much smaller than for a whole population” (Rust and Golombok, 1998, p. 68). In fact, available data show that attitudes toward IPVAW are modulated by individual, organizational, community, and social factors (Flood and Pease, 2009; Waltermaurer, 2012), including gender but also age or educational level (Brandt, 2011; Herrero et al., 2017), although previous research yields contradictory results (Flood and Pease, 2009; Wang, 2016; Herrero et al., 2017). But despite the relevance of these factors, the vast majority of available research focuses on young people, and in particular on university students (Gracia et al., 2020).

Additionally, gender was determined by a single self-report item in a brief questionnaire designed *ad hoc* (following previous studies, e.g., Lehavot et al., 2012). However, it may be possible that a single self-categorization item was not enough. So, future studies should consider administering multi-item scale measures to assess the participants’ gender self-categorization.

Moreover, “the effect of social desirability should be carefully examined in future studies, assessing the relationship between (…) self-reported measures and implicit measures of acceptability of IPVAW” (Martín-Fernández et al., 2018, p. 31). In any case, the discrepancy between implicit and explicit measures cannot be explained solely by factors of social desirability or deliberate attempts at concealment but, rather, by the multiple personal, interpersonal, contextual, and methodological moderating factors involved (Nosek, 2005; Hofmann et al., 2005; Gawronski and Bodenhausen, 2017; Gawronski and Hahn, 2019; Kurdi et al., 2019). For instance, as Martín-Fernández et al. (2018) point out, future research should determine whether gender differences encountered in IPVAW attitude measures are in fact “due to actual differences between latent means for men and women, and not to different interpretations of the items” (p. 31. Given that self-reports regarding attitudes toward sensitive topics such as IPVAW may be biased, it is important to complement the exploration of these attitudes with alternatives such as indirect or implicit measures (Fazio and Olson, 2003; Eckhardt et al., 2012). In other words both explicit and implicit measures must be utilized. The results obtained and presented here provide additional support to the idea that GV-IAT constitutes a promising assessment tool to complement explicit measures for attitudes toward IPVAW.

## Data Availability Statement

The raw data supporting the conclusions of this article will be made available by the authors, without undue reservation.

## Ethics Statement

The studies involving human participants were reviewed and approved by Ethics Research Committee (University of Balearic Islands, Spain). The patients/participants provided their written informed consent to participate in this study.

## Author Contributions

VF-P, EB-F, VF-B, CD-A, and AS-P conceived and designed the research, performed the research, wrote the manuscript, and approved the final manuscript. CD-A and AS-P analyzed the data. VF-P and EB-F revised the article critically. All authors contributed to the article and approved the submitted version.

## Conflict of Interest

The authors declare that the research was conducted in the absence of any commercial or financial relationships that could be construed as a potential conflict of interest.

## Footnotes

1 https://bkurdi.shinyapps.io/reliCalc/
